# Crop management as a driving force of plant growth promoting rhizobacteria physiology

**DOI:** 10.1186/s40064-016-3232-z

**Published:** 2016-09-15

**Authors:** Juliana Melo, Manuela Carolino, Luís Carvalho, Patrícia Correia, Rogério Tenreiro, Sandra Chaves, Ana I. Meleiro, Sávio B. de Souza, Teresa Dias, Cristina Cruz, Alessandro C. Ramos

**Affiliations:** 1 Ecosystems Ecology Unit, Universidade Vila Velha (UVV), Vila Velha, ES 29102-920 Brazil; 2 Center for Ecology, Evolution and Environmental Changes, Faculdade de Ciências, Universidade de Lisboa, 1649-004 Lisbon, Portugal; 3 Center for Biodiversity, Functional and Integrative Genomics, Faculdade de Ciências, Universidade de Lisboa, 1649-004 Lisbon, Portugal; 4 Physiology and Biochemistry of Microorganisms Lab., Center of Biosciences and Biotechnology, Universidade Estadual do Norte Fluminense Darcy Ribeiro (UENF), Rio de Janeiro, 28013-620 Brazil

**Keywords:** Biocontrol, Organic farming, Conventional farming, Fungi, Nitrogen fixation, Rhizosphere

## Abstract

Crop management systems influence plant productivity and nutrient use efficiency, as well as plant growth-promoting rhizobacteria (PGPR), which are known to influence the growth of plants via phytohormone production, phosphate solubilization, nitrogen (N) fixation and antimicrobial activity. The objective of this study was to compare the influence of two crop management system on microbial PGPR features. PGPR isolated from the rhizospheres of *Carica papaya* L. grown under two distinct management systems (conventional and organic) were identified and characterized. The 12 strains most efficient in solubilizing inorganic phosphate belonged to the genera *Burkholderia*, *Klebsiella*, and *Leclercia.* N fixation was observed in the strains *B. vietnamiensis* from the conventional farming system and *B. vietnamiensis*, *B. cepacia* and *Leclercia* sp. from the organic farming system. The *B. vietnamiensis*, *B. cepacia*, *Klebsiella* sp. and *Klebsiella* sp. isolates showed antifungal activity, while *Leclercia* sp. did not. The strains *B. vietnamiensis* and *Enterobcter* sp. (isolated from the conventional farming system) and *Klebsiella* sp. (isolated from the organic farming system) were efficient at solubilizing phosphate, producing phytohormones and siderophores, and inhibiting the mycelial growth of various phytopathogenic fungi (*Botrytis cinerea*, *Pestalotia* sp., *Alternaria* sp., *Phoma* sp., *Fusarium culmorum*, *Geotrichum candidum*). Physiological differences between the isolates from the two crop management regimes were distinguishable after 10 years of distinct management.

## Background

Agriculture is struggling to meet the enormous challenge of producing enough food for an ever-expanding world population. To maintain this high productivity, great quantities of synthetic fertilizers are required (Avis et al. 2008; Godfray et al. 2010), which can damage ecosystem structures and functions, including the soil microbial community which plays an important role in agriculture sustainability (Ahemad and Khan 2010; Avis et al. 2008; Srinivas et al. 2008).

The structure and function of the soil microbial community are directly and indirectly influenced by the management regime (Barea et al. 2005; Araújo et al. 2009). On one hand, intensive conventional farming systems are dependent on large fertilizer inputs and characterized by low nutrient use efficiencies, which result in environmental threats (Marinari et al. 2010; Radíc et al. 2014) including environmental pollution, soil erosion (Marja et al. 2014) and loss of biodiversity (Dai et al. 2014). On the other hand, organic farming is defined as sustainable since it relies on diversified inputs, most of them organic and not immediately available to the crop, which stimulate the networking and complementarity of the soil microbial community, and reduce environmental threats (Beltrán-Esteve and Reig-Martínez 2014; Marja et al. 2014; Perez et al. 2014). As nitrogen (N) and phosphorus (P) are the main fertilizers used worldwide, and have biogeochemical cycles involving many bio-transformations, the efficiency of their use by plants is intrinsically related with the crop management regime (van Diepeningen et al. 2006; Moeskops et al. 2010; Barea et al. 2014). Many of the plant growth promoting rhizobacteria used as biofertilizers are involved in the soil P and/or N cascades of transformation (Chiarini et al. 1998).

PGPR constitute a heterogeneous group that include organisms of the genera *Pseudomonas*, *Azospirillum*, *Burkholderia*, *Bacillus*, *Enterobacter*, *Klebsiella*, *Rhizobium*, *Erwinia*, *Serratia*, *Alcaligenes*, *Arthrobacter*, *Acinetobacter*, and *Flavobacterium* (Rodríguez and Fraga 1999; Khan et al. 2009; Sharma et al. 2013; Ahemad and Kibret 2014; Reed et al. 2015).

PGPR exert beneficial effects on plant growth via direct and indirect mechanisms. Direct mechanisms involve the synthesis of substances or modulation at an enzymatic level, which facilitates the absorption of certain nutrients, solubilization of mineral phosphates (Ahemad et al. 2008; Malboobi et al. 2009), biological fixation of nitrogen (Peix et al. 2003; Caballero-Mellado et al. 2007; Jackson et al. 2008) and synthesis of plant hormones such as gibberellic acid, cytokinins, ethylene, and indolic acetic acid (IAA). Indirect mechanisms include PGPR decreasing or preventing the destructive effects of one or more phytopathogens, by the production of antibiotics (Richardson et al. 2009; Bevivino et al. 1994; Rodríguez and Fraga 1999) or siderophores (Reed et al. 2015). Siderophores produced by PGPR have a high affinity with iron III from the rhizosphere and, consequently, retain a most of the iron available, inhibiting the proliferation of phytopathogenic fungi (Bevivino et al. 1998; Laslo et al. 2012).

However, sometimes the effects of PGPR are unstable, and greatly influenced by biotic and abiotic soil conditions, conferring on PGPR a certain degree of specificity in relation to crop improvement. It is consequently recommended that, in order to obtain the best results, PGPR inoculants should be isolated from native PGPR populations (Reed et al. 2015; Santos-Villalobos et al. 2012; Bashan et al. 2014). This poses the question of what is the bigger driver for the rhizobacteria’s functional ecology: the crop or management regime? This work was intended to address this question by studying potential PGPR isolated from the rhizosphere of papaya (*Carica papaya* L.) plants grown in farms with similar soil and edaphic conditions, under distinct crop management regimes.

Papaya is one of the most commonly cultivated fruits in almost all tropical American countries, and it is also one of the most consumed fruits in the tropical and sub-tropical regions of the world (FAO 2012). Due to its continuous growth, uninterrupted and simultaneous flowering and fruiting, it needs water and nutrients throughout its growing cycle (Mendonça et al. 2006; Trindade et al. 2006) and therefore is a high nutrient-demanding crop. This work is based on the hypothesis that the different crop management systems represent distinct soil perturbations and influence differently the microbial structure and the mechanisms involved in the promotion of plant growth. The objective of this study was to compare the influence of two crop management system (conventional and organic) on microbial PGPR features.

## Results

### Isolation and identification of phosphate-solubilizing bacteria using 16S rRNA sequencing

The shortage of P reserves and their rapid loss in the soil even after fertilization promotes the search for PGPR with phosphorus solubilizing capacity which may eventually be used as soil inoculants. Therefore our initial aim was to conduct a screening for selecting bacteria isolates with high ability to solubilize phosphate.

**Fig. 1:**
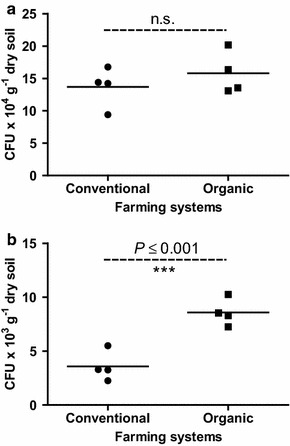
Bacterial total density (**a**) and tricalcium phosphate solubilizing bacterial Density (**b**) in the rhizosphere of *C*. *papaya* L. plants grown in conventional or organic system (n = 4). n.s. means are not significantly by Student’s *t* test at P ≤ 0.05. *** Means are statistically different by Student’s *t* test at P ≤ 0.001

The counts of total cultivable bacteria isolated from the rhizosphere of *C. papaya* were not influenced by the management regime (Fig. 1a). However, the number of rock phosphate-solubilizing bacteria (based on the CFU formed in the selective medium) was higher in soils from the organic system (P = 0.02) (Fig. 1b).

**Fig. 2:**
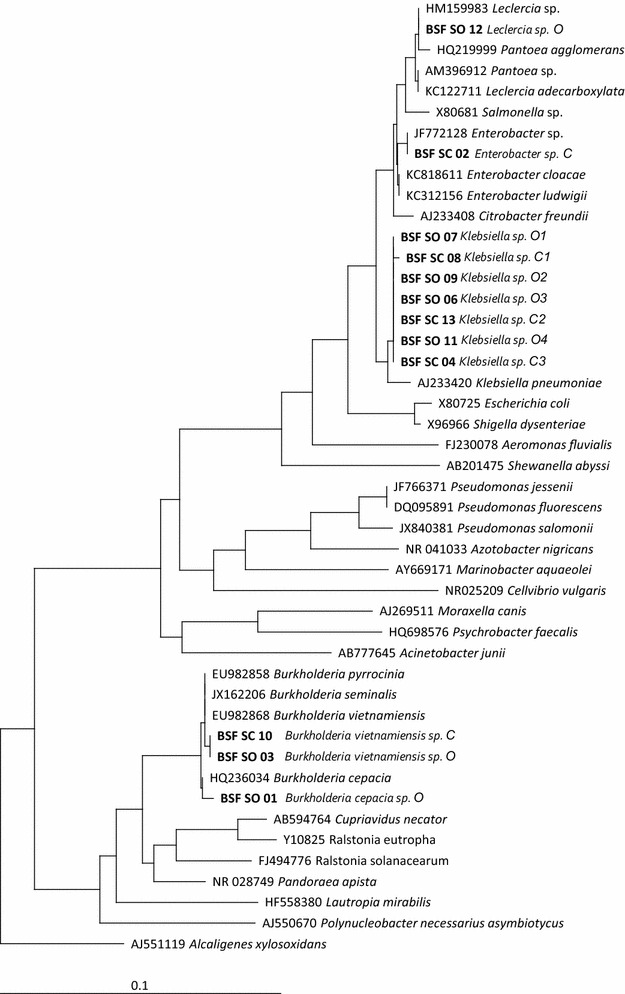
Phylogenetic tree obtained with 16S rDNA partial sequences (518 nucleotide positions), corresponding to the PSB isolates and the most closely related ones retrieved from BLAST search. Phylogeny was inferred using the Neighbor Joining method of aligned 16S rDNA fragments. *Alcaligenes xylosoxidans*, was included to root the tree. Access numbers of GenBank sequences are indicated in the figure and names in *bold face* correspond to sequences determined in this work

The 12 CFUs presenting the greater rock phosphate-solubilizing capacity (higher solubilization index), in the NBRIP medium, 5 from the conventional and 7 from the organic farming systems, were selected for molecular identification. All the isolates preserved in vitro conditions, maintained their viability and functionality over time in laboratory assays). The 12 isolates were identified as belonging to two families: Enterobacteriaceae and Burkholderiaceae. Seven isolates were identified as belonging to the genus *Klebsiella*, the predominant genus in both management systems and were very similar to each other. The others were of the genera *Burkolderia* (3, including one *B. cepacia* and two *B. vietnamiensis*), *Enterobacter* (1) and *Leclercia* (Fig. 2).

The five isolates from the conventional farming system were identified as *Enterobacter* sp., *Klebsiella* sp. and *Burkholderia vietnamiensis*, designated in this work as *Enterobacter* sp. C, *Klebsiella* sp. C1, *Klebsiella* sp. C2, *Klebsiella* sp. C3 and *Burkholderia vietnamiensis* C. The seven isolates from the organic farming system were *Burkholderia cepacia*, *Burkholdeira vietnamiensis*, *Klebsiella* sp. and *Leclercia* sp., here designated *Burkholderia cepacia* O, *Burkholderia vietnamiensis* O, *Klebsiella* sp. O1, *Klebsiella* sp. O2, *Klebsiella* sp. O3, *Klebsiella* sp. O4 and *Leclercia* sp. O (where O stands for the organic management system).

### Solubilization index (SI) for different phosphate sources and potential plant growth promoter

**Fig. 3:**
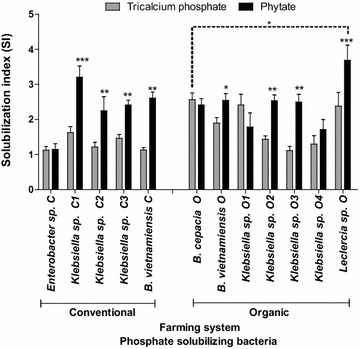
Phosphate solubilization index in solid NBRIP supplemented with tricalcium phosphate or phytate by phosphate solubilizing bacteria isolated from conventional and organic farming systems (n = 4). Means are statistically different by Student’s *t* test (*P ≤ 0.05; **P ≤ 0.01; ***P ≤ 0.001)

Although the isolates from the organic system had the highest capacity to solubilize rock phosphate, the differences between the farming systems were not so clear in relation to their capacity to use phytate (Fig. 3), the main organic source of phosphorus in the soil, as a P source. Of the twelve isolates, only four (*B. vietnamiensis* C, *B. vietnamiensis* O, *B. cepacia* O and *Leclercia* sp O) displayed the ability to fix nitrogen. Siderophore production was observed in all the bacterial isolates. With the exception of isolated *Leclercia* sp O, With the exception of *Leclercia* sp O, all the other strains were resistant to ampicillin and methacycline, and were susceptible to chloramphenicol, kanamycin, erythromycin and tetracycline.

### Temporal kinetics of IAA production

**Fig. 4:**
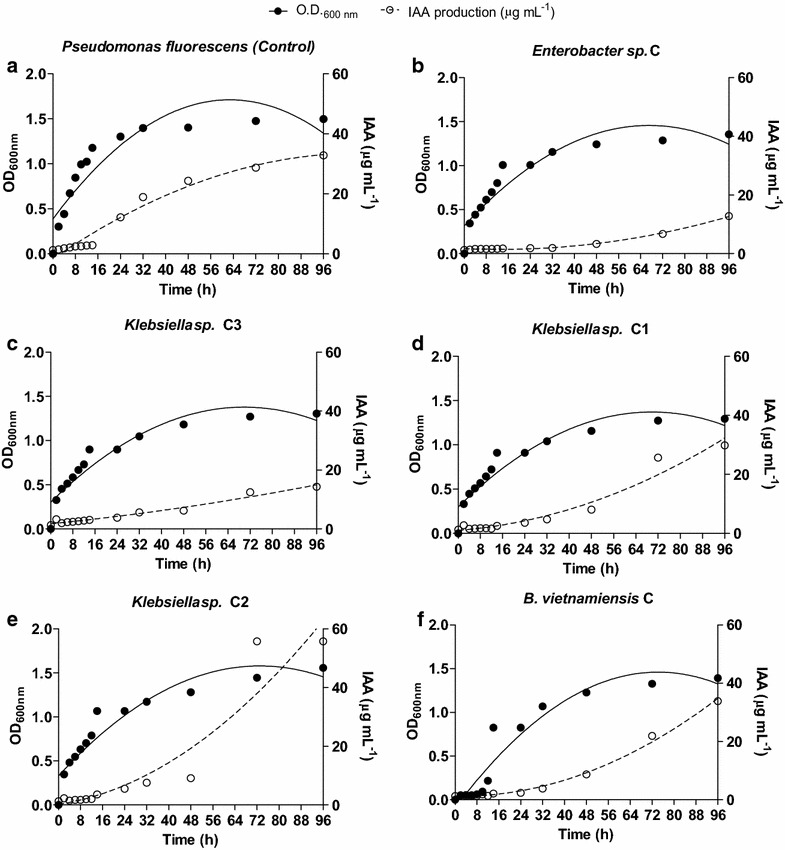
IAA production kinetics and bacterial growth of *Pseudomonas fluorescens* (**a**), *Enterobacter sp.* C (**b**), *Klebsiella sp.* C3 (**c**), *Klebsiella sp.* C1 (**d**), *Klebsiella sp.* C2 (**e**), *B. vietnamiensis* C (**f**) isolated from the rhizosphere of *C*. *papaya* L. (conventional farming system) (n = 4)

**Fig. 5:**
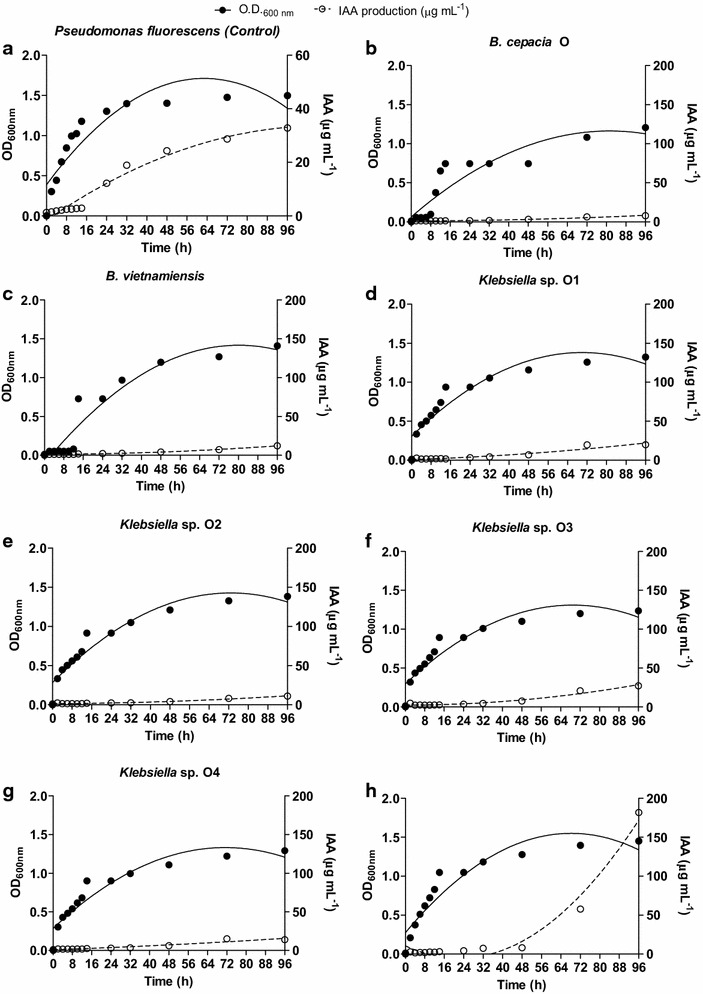
IAA production kinetics and bacterial growth of *Pseudomonas fluorescens* (**a**), *B. cepacia* O (**b**), *B. vietnamiensis* (**c**), *Klebsiella* sp. O1 (**d**), *Klebsiella* sp. O2 (**e**), *Klebsiella* sp. O3 (**f**), *Klebsiella* sp. O4 (**g**) and *Leclercia* sp. O (**h**) isolated from the rhizosphere of *C*. *papaya* L. (organic farming system) (n = 4)

Regardless of the farming system, the highest production of IAA by all the isolated bacteria (in the culture medium supplemented with 200 ng mL^−1^ of tryptophan) took place at the beginning of the stationary phase of bacterial growth (Figs. 4, 5).

The colonies assessed in this study presented the largest production of IAA after 96 h of incubation, with an optical density (OD) of 1.3 (Figs. 4, 5). IAA production by the bacterial colonies from the conventional farming system ranged from 15 to 60 µg mL^−1^ (Fig. 4). Of these, the lowest IAA production observed was that by *Enterobacter* sp C (15 µg mL^−1^), with an OD of 1.3 (Fig. 4b). The highest production (60 µg/mL) was that by *Klebsiella* sp C2, also with an OD of 1.3 after 96 h (Fig. 4d) of incubation. After 96 h, the other isolates from this farming system (*Klebsiella* sp C1, *B.* *vietnamiensis* C and *Klebsiella* sp C3) produced 30, 35 and 20 µg mL^−1^ of IAA, respectively. The OD was also 1.3 (Fig. 4c, e, f).

The production of IAA by isolates from the organic farming system after 96 h ranged from 8 to 180 µg mL^−1^, the OD being 1.3 (Fig. 5). The lowest production observed was that by *B. cepacia* O (8 µg mL^−1^) (Fig. 5b), while the highest was that by *Leclercia* sp. O (180 µg/mL) (Fig. 5h). The colonies of the other isolates (*B. vietnamiensis* O, *Klebsiella* sp. O1 *Klebsiella* sp. O2, *Klebsiella* sp. O3 and *Klebsiella* sp. O4) presented, after 96 h, IAA productions of 14, 20, 12, 27 and 14 µg/mL respectively, with an OD of 1.3 (Fig. 5b–g).

**Fig. 6:**
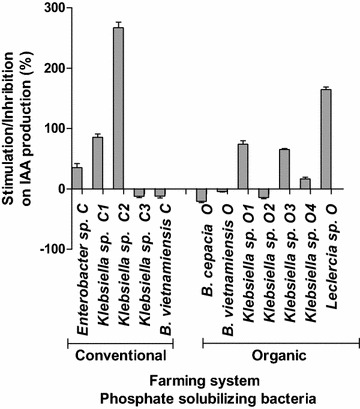
Inhibition and stimulation in the production of IAA in medium supplemented with 2 (control) and 200 mg L^−1^ of tryptophan by bacteria isolated from the rhizosphere of *C*. *papaya* L. in conventional and organic farming systems (n = 4)

All the strains isolated from the rhizosphere of *C. papaya* except *Klebsiella* sp. C1, *B. vietnamiensis* C and *Klebsiella* sp. O2, *B. vietnamiensis* O and *B.* *cepacia* O, increased production of IAA in the presence of 200 μg mL^−1^ of the precursor tryptophan, in comparison to the control with 2 μg mL^−1^ of tryptophan (Fig. 6).

### Antifungal activity

**Fig. 7:**
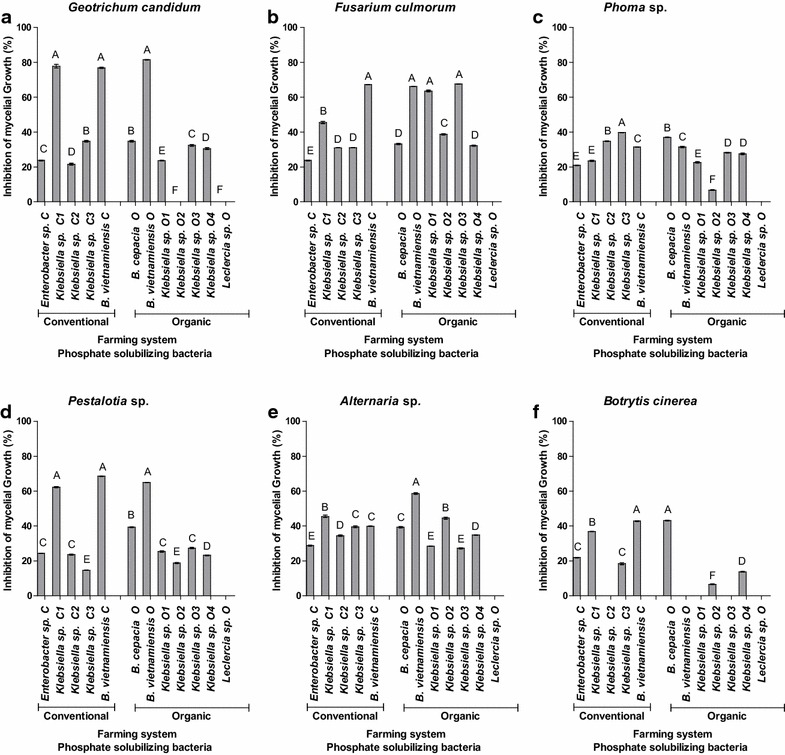
Inhibition of mycelium growth rate of *Geotrichum candidum* (**a**), *Fusarium culmorum* (**b**), *Pestalotia* sp. (**c**), *Alternatia* sp (**d**), *Phoma* sp. (**e**) and *Botrytis cinerea* (**f**) by bacteria isolated from the rhizosphere of *C*. *papaya* L. (organic and conventional farming system (n = 4). Mean values followed by the same capital letters do not differ significantly by the Tukey test (P ≤ 0.05)

The isolates displayed antifungal properties against the phytopathogenic fungi *Fusarium culmorum*, *Geotrichum candidum*, *Pestalotia sp.*, *Alternaria sp.*, *Phoma sp.* and *Botrytis cinérea* (Figs. 7). Which are associated with papaya culture, for instances *Fusarium culmorum*, *Phoma* sp. and *Alternaria* sp induce peduncular rot in Papaya (Suzuki et al. 2007; Nery-Silva et al. 2007) seriously affecting papaya export and farmers income. The most effective isolates tested were *Klebsiella* sp. C1 and *B. vietnamiensis* C, which inhibited mycelial growth by 78 and 76 % respectively. *B. vietnamiensis* O inhibited mycelial growth of *Geotrichum candidum* by 81 % (Fig. 7a). Inhibition of mycelial growth of *Fusarium culmorum* by the isolates was over 30 %, the greatest inhibition being by *B. vietnamiensis* C (67 %), *Klebsiella* sp. O1, (68 %), *B. vietnamiensis* O (66 %) and *Klebsiella* sp. O3 (63 %) (Fig. 7b).

The greatest inhibition of mycelial growth of the fungus *Pestalotia* sp. was by the isolates *B. vietnamiensis* C (68 %) and *Klebsiella* sp. C1 (62 %) (Fig. 7c). The phytopathogenic fungus *Alternaria* sp. was inhibited by more than 30 %. The greatest inhibition was by *Klebsiella* sp.C1 (45 %), *B. vietnamiensis* O (58 %) and *Klebsiella* sp. O2 (44 %) (Fig. 7a). *Phoma* sp., was inhibited by up to 40 %, the greatest inhibition being by *Klebsiella* sp. C3 (40 %) and *B. cepacia* O (37 %) (Fig. 7b).

All the bacterial isolates inhibited the phytopathogenic fungus *Botrytis cinerea* by less than 44 % and the greatest inhibition was that by *B. vietnamiensis* C, *B. cepacia* O and *B.* *vietnamiensis* O (Fig. 7c).

## Discussion

The crop management regime did not affect the size of the culturable bacteria community in the rhizosphere. However, it did affect the size and structure of the bacterial community able solubilize rock phosphate (Figs. 1, 2). The effect of the management system on bacterial numbers is controversial. Some studies did not observe differences (Shannon et al. 2002), while others found higher numbers of bacteria in the rhizospheres of organically grown crops (Grantina et al. 2011). It is possible that part of the variation can be explained by soil pH and chemical composition, as well as by the quantities and nature of the fertilizers applied (Naher et al. 2013). The effect of the management system on certain bacterial functional groups (such as the phosphate-solubilizing bacteria) may be direct or indirect. In our study the higher number of rock phosphate-solubilizing bacteria observed in the soils of the organic farming system may be due to the soil pH (6.5), which is more suitable for the functionality of phosphate-solubilizing bacteria than the 5.5 observed in the soils of the conventional system (Table 1). On the other hand, the phosphate fertilizer applied to the conventional system may inhibit phosphate solubilization (Naher et al. 2013).

**Table 1:** Chemical properties of a *C*. *papaya* L. rhizospheric soil under both conventional and organic farming systems

Chemical characterization	Conventional	Organic	P value
pH	5.5	6.6*	0.041
P (mg dm^−3^)	355.3	382.7^n.s.^	0.643
K (mg dm^−3^)	189.0	116.0*	0.032
Ca (cmolc dm^−3^)	4.4	4.4^n.s.^	0.812
Mg (cmolc dm^−3^)	0.6	0.9^n.s.^	0.073
Na (cmolc dm^−3^)	0.1	0.2^n.s.^	0.056
C (%)	1.4	1.3	0.104
MO (g dm^−3^)	23.3	22.4^n.s.^	0.138
Cu (mg dm^−3^)	2.8	8.6***	0.00014
Zn (mg dm^−3^)	22.6	14.0**	0.0085
Mn (mg dm^−3^)	10.0	23.0**	0.0012
S (mg dm^−3^)	127.5	35.3***	0.00001
B (mg dm^−3^)	0.9	1.4*	0.0193
Fe (mg dm^−3^)	26.8	21.2*	0.045
H + Al (cmolc dm^−3^)	2.3	0.8*	0.014
BS (cmolc dm^−3^)	5.6	5.7^n.s.^	0.569
T (cmolc dm^−3^)	7.9	6.5*	0.027
t (cmolc dm^−3^)	5.6	5.7^n.s.^	0.452
V (%)	70.7	87.3*	0.017

H + Al, potential acidity; BS, sum of bases; T, cation exchange capacity (CEC) at pH 7.0; t, effective cation exchange capacity (eCEC); V, saturation percentage for bases; ^n.s^, means are not significantly different by Student’s t test at P ≤ 0.05

* Means are statistically different by Student’s t test at P ≤ 0.05, ** P ≤ 0.01 and *** P ≤ 0.001

Molecular identification of the isolates showed that they belong to the genera *Burkholderia*, *Enterobacter*, *Klebsiella* and *Leclercia*, the first three of which include many known PGPR (Rodríguez and Fraga^1999^; Madhaiyan et al. 2006). Many strains of *Burkholderia cepacia* have already been described as PGPR (Dawwam et al. 2013; Di Cello et al. 1997; Rodríguez et al. 2006; Pereg and McMillan^2014^), others are known as opportunistic pathogens in patients with cystic fibrosis and other immune depressive diseases (Richardson et al. 2009; Bhardwaj et al. 2014; Peeters et al. 2013). However, Bevivino et al. (1994) claimed that strains isolated from different sources show genetic differences and differ in their degree of pathogenicity. In fact, some strains of *B. cepacia* are already being used in the biological control of plant pathogens (Bevivino et al. 1994; Huang et al. 2013). Based on the identification of these 12 isolates, we could not determine any structural differences between the phosphorus-solubilizing communities of the two management systems’ soils. One genus was only identified in soils from the conventional system (*Enterobacter*), while another was only detected in soils from the organic system (*Leclercia*, Fig. 2) However most of the isolates from soils of both management systems belonged to the genus *Klebsiella*.

The capacity to solubilize inorganic phosphate is a necessary characteristic of a PGPR, however in most soils phytate, which is not assimilated by plants, is the largest soil P pool (Singh et al. 2014). In this study, all 12 bacterial isolates from soils of both systems showed a high capacity to solubilize phytate (Fig. 3). Similar results were obtained in the rhizosphere of *Lolium perenne* L., *Trifolium repens* L., *Triticum aestivum* L., *Avena sativa* L. (Jorquera et al. 2008); *Lupinus luteus* L. (Unno et al. 2005) or *Lupinus albus* (Patel et al. 2010; Hayat et al. 2010). The capacity to mineralize phytate is important: in *B. amyloliquefaciens* it positively correlates with the promotion of corn seedlings’ growth (Idriss et al. 2002). Thus, mineralizing microorganisms capable of promoting phytate use in the rhizosphere have been considered for application as biofertilizers (Unno et al. 2005; Jorquera et al. 2008; Singh et al. 2014). In contrast to phytate mineralization, isolates from the organic farming system were better able to solubilize tricalcium phosphate than those from the conventional system.

It is generally agreed that bacteria with a high capacity to solubilize phosphorus also exhibit diazotrophic function (Araújo et al. 2009). However, only 4 of the 12 isolates obtained in this study were diazotrophic: 3 from the organically-managed plot and 1 from the conventional management system (Table 2). The four isolated strains with high phosphorus solubiization capacity and the ability to fix nitrogen were *B. cepacia* O, *B. vietnamiensis* O, *Leclercia* sp O, and *B. vietnamiensis* C. As expected and reported by other authors, the *Enterobacter* isolate was not diazotrophic. The increase in plant nitrogen acquisition usually associated with *Enterobacter* PGPR may be related with changes in the plant hormonal balance induced by the bacteria (Martínez-Aguilar et al. 2008; Azadeh et al. 2010; Ferrara et al. 2012). The *Klebsiella* isolated did not display nitrogen fixation, although some species of these genus are diazotrophic (Rogers et al. 2011).

**Table 2:** Physiologic characteristics and antibiogram for the phosphate solubilizing bacteria isolated from rhizosphere of *C. papaya* L. in both conventional and organic farming systems

	Farming system											
	Conventional					Organic						
	*Enterobacter sp* C	*Klebsiella* sp C1	*Klebsiella* sp C2	*Klebsiella* sp C3	*B. vietnamiensis* C	*B.cepacia* O	*B. vietnamiensis* O	*Klebsiella* sp O1	*Klebsiella* sp O2	*Klebsiella* sp O3	*Klebsiella* sp O4	*Leclercia* sp O
Physiology												
N Fixation	*	*	*	*	++	++	++	*	*	*	*	++
Siderophore Production	+	+	+	+	+	+	+	+	+	+	+	+
Antibiotic Resistance												
Metacycline	r	r	r	r	r	r	r	r	r	r	r	nd
Ampicillin	r	r	r	r	r	r	r	r	r	r	r	nd
Chloramphenicol	s	s	s	s	s	s	s	s	s	s	s	nd
Erythromycin	s	s	s	s	s	s	s	s	s	s	s	nd
Kanamycin	s	s	s	s	s	s	s	s	s	s	s	nd
Tetracycline	s	s	s	s	s	s	s	s	s	s	s	nd

*, no nitrogen-fixing; ++, nitrogen-fixing; +, producing Siderophores; r, antibiotic resistant; s, susceptible to antibiotics; nd, not detectable

All strains isolated were able to produce siderophores, a frequent characteristic of PGPR (Richardson et al. 2009; Gupta et al. 2012; Loaces et al. 2011, Table 2). Siderophores are low molecular weight peptides or iron chelators which are synthesized by many microorganisms (Vassilev et al. 2006; Patel et al. 2010; Santos-Villalobos et al. 2012) and some plant species. The types of siderophores produced vary according to the microbial functional group (Lugtenberg and Kamilova 2009). Microorganisms capable of producing siderophores are beneficial to plants because they increase iron availability to the plant (Lugtenberg and Kamilova 2009; Ahmed and Holmström 2014), and perhaps because they are related with increased synthesis of antifungal compounds, thus providing protection against pathogens (Davidson 1988; Laslo et al. 2012).

 So far no bacterial physiological role has been attributed to IAA. However many bacteria produce high amounts, especially when in the presence of tryptophan (Amin et al. 2015; Zúñiga et al. 2013). It has been suggested that IAA production by bacteria may be part of a signaling network between bacteria and non-bacterial partners. In the case of the interaction between PGPR and plants, this chemical crosstalk is very important, since IAA is a phytohormone controlling many important physiological processes in plants, such as cell division, tissue differentiation, root initiation, and growth (Khan et al. 2014). IAA accumulation was observed during the later stages of growth, after the stationary growth phase, which is characteristic of secondary metabolite production (Unno et al. 2005; Tsavkelova et al. 2005; Richardson et al. 2009; Ahemad and Khan 2011; Gupta et al. 2012; Figs. 4, 5). As expected (Tsavkelova et al. 2005), the highest production of IAA was observed in the presence of 200 mg L^−1^ of tryptophan (Fig. 6). Considering that root exudates of many plants are rich in tryptophan (Tsavkelova et al. 2005; Singh et al. 2013), the chemical network between plants and PGPR which produce IAA is obvious.

All the isolates except *Leclercia* sp O inhibited the mycelial growth of the phytopathogenic fungi *Geotrichum candidum*, *Fusarium culmorum*, *Pestalotia* sp, *Alternaria* sp., *Phoma* sp and *Botrytis cinerea* (Fig. 7). The antifungal activity of *B. cepacia* has also been observed by other authors and was correlated with the fungus’ ability to produce siderophore peptides (Bevivino et al. 1998; Trujillo et al. 2007; Ahemad and Khan 2010). Some *Enterobacter* strains achieve suppression of mycelial growth of various phytopathogenic fungi through high activities of chitinolytic enzymes (Chernin et al. 1995).

Based on the sequencing of 16 s rRNA, all the isolates identified as belonging to the *Klebsiella* genus were genetically very similar. However, they presented distinct physiological profiles in relation to their capacity to solubilize rock phosphate, produce IAA and inhibit the growth of phytopathogenic fungi. These distinct phenotypes may correspond to distinct genotypes (not detected by the 16 s rRNA sequencing) or distinct gene expression due to environmental factors (Buckling et al. 2003; Bochner 2009).

## Conclusions

The objective of this study was to compare the influence of two crop management (conventional and organic) in several important PGPB features.

In this study it was observed that the crop management regime under which *C. papaya* was grown influenced the physiological functions the strains isolated from their rhizospheres, most notably inorganic phosphate solubilization and nitrogen fixation. Although most strains isolated in this study showed high potential to improve plant growth and be used as PGPR, highlighting the importance of assessing the pathogenicity and opportunistic behavior of isolates. The isolated strains *B. vietnamiensis* C, *B. vietnamiensis* O, *Klebsiella* sp. O1 and *Enterobacter* sp. C clearly showed the capacity to solubilize phosphate, produce phytohormones and siderophores, and inhibit the growth of phytopathogenic fungi in vitro, and therefore can be considered as promising candidates to be used in biofertilizers. However, more studies are needed to understand how these isolates behave when used to inoculate plants, and evaluate the performance of plants inoculated under field conditions.

Although no substantial difference was found in several PGPR traits (IAA and siderophore production, nitrogen fixation and antifungal activity against plant pathogenic fungi) our results revealed that as a group, the isolates from the organic farming system, showed a higher potential efficiency to solubilize inorganic phosphates than the group from the conventional crop. However, more studies are needed to understand the effect of crop management on the structure of the bacterial community, including a metagenomic analysis using taxonomic and functional genetic markers.

## Methods

### Sample description and chemical analysis of the rhizospheric soil

The study was carried out in an area where *C. papaya* L. (Golden variety) is grown under two different management regimes (conventional and organic), in Sooretama, in Espírito Santo state, Brazil, between 2012 and 2013. The organic area has quality certifications: in 2004 it obtained the “EUREPGAR Protocol for Fresh Fruits and Vegetables” certificate and in 2009 the organically-farmed area obtained the IBD organic certificate (Inspeções e Certificações Agropecuárias e Alimentícias). Local average annual temperature and precipitation are 22 °C and 700–800 mm. The experimental areas have a plain topography and a deep soil classified as moderate type A Dystrophic Yellow Podzolic with a sandy/medium texture. The plantation was carried out in planting holes (40 × 40 × 40 cm^3^), with a volume of 64 L, with gaps of 3.0 × 2.0 m in a simple row system resulting in 1700 plants/ha^−1^. Each planting hole was fertilized.

The conventional farming system (19°12′22.9″S 40°05′52.0″W) had been under natural vegetation until 1994, and was then used to grow conventional conillon coffee plants. Since 2004 *C. papaya* has been cultivated conventionally, using industrial chemicals for soil fertilization. Chemical pesticides are used for pest control and the soil irrigation is carried out by micro-sprinklers.

The organic farming system (19°14′13.40″S 40°4′38.73″W) was also under natural vegetation until 1994 and was also used to grow conventional conillon coffee plants, however, this crop was abandoned in 1999. During the year 2000, the plants in question were removed and the soil was then rested until 2004, allowing the grass to grow. In 2005, cultivation of *C. papaya* started with monthly soil fertilizations using liquid fertilizers with the amino acids Turbofil formulation: wood dirt, filter cake, natural phosphates, magnesium oxide, dolomite limestone, bone flour, fresh cow manure, molassed chicken manure, and ground ox liver. All the ingredients were mixed and left to rest for 40 days before being used. Double ventilated sulphur powder was used for the control of white mites, whilst cassava water was used every week to control other pests. Soil irrigation was carried out by micro-sprinklers.

Rhizospheric soil samples were collected at 0–20 cm depth, following EMBRAPA soil collection protocols (EMBRAPA 2005). Each sampled soil parcel contained 6 plants spaced at a distance of 20 cm. Four soil subsamples were collected from around each plant, at 8 cm from the plant neck. Soil samples were collected in labelled plastic bags and placed in polystyrene boxes that kept soil temperature constant, then transported to the laboratory, where they were immediately analysed.

For chemical analysis, a portion of each sample was dried in an incubator with forced air circulation at 60–70 °C for 24 h. After drying, samples were ground and sieved (2 mm) before storage in vacuum sealed plastic containers for further analysis (Sarruge and Haag 1974; Martins and Reissmann 2007). P was determined colorimetrically based on the ammonium vanadate-molybdate method. K, Al and Na were measured by flame photometry. Ca, Mg, S, Cu, Fe, Mn, B and Zn were determined by atomic absorption spectrophotometry (Table 1).

### Isolation of total bacteria

Total culturable bacteria were isolated by serial dilution in a homogeneous suspension of 10 g soil in 90 mL of saline solution (0.85 % NaCl), shaken at 250 rpm for 30 min at 25 °C. Dilutions from 10^−2^ to 10^−6^ were plated on solid medium Nutrient Agar, and incubated for 5 days at 28 °C. Data were expressed as number of colony forming units (CFU) per g of dry soil (Grantina et al. 2011).

### Isolation of phosphate-solubilizing bacteria

Phosphate-solubilizing bacterial strains were isolated by serial dilution as described for total bacteria, but the 10^−2^ and 10^−6^ dilutions were smeared into solid medium (National Botanical Research Institute’s phosphate (NBRIP) (Nautiyal 1999) containing 10 g of glucose, 5 g of Ca_3_(PO_4_)_2_, 5 g of MgCl_2_·6H_2_O, 0.25 g of MgSO_4_·7H2O, 0.2 g of KCl, 0.1 g of (NH_4_)_2_SO_4_ and 1.5 % of agar in 1 L deionised water (pH 7.0). After incubation for 15 days at 28 °C, bacteria that presented a clear halo around the colony were considered capable of solubilizing tricalcium phosphate. Their numbers were expressed as colony forming units (CFU) per gram (g) of dried soil. Selected strains were replicated into new plates containing NBRIP solid medium. After the second passage, the isolated colonies were stored in DYGS culture media containing 2 g of glucose, 1.5 g of peptone, 2 g of yeast extract, 0.5 g of KH_2_PO_4_·7H_2_O and 0.5 g of MgSO_4_·7H_2_O in 1 L deionised water (pH 6.8). They were subsequently stored in 50 % glycerol at −80 °C.

### Molecular characterization of isolates

#### DNA extraction

Genomic DNA from all studied isolates was extracted following the method described by (Pitcher et al. 1989), with some modifications. Briefly, bacterial cells were collected by centrifugation and washed with TE buffer. The pellets were then re-suspended in 500 μL TE and digested with 250 μg lysozyme for 1 h at 37 °C. 500 μL of GES (5 M guanidium tiocyanate, 100 mM EDTA, 0.5 % (v/v) sarcosil) were added. The suspension was incubated on ice until cell lysis, then 250 μL of 10 M ammonium acetate were added and the suspension was re-incubated on ice for 10 min. Nucleic acids were extracted with chloroform:isoamilic alcohol (24:1) and precipitated with one volume of isopropanol. After centrifugation, DNA was washed with ethanol 70 % (v/v), resuspended in TE buffer with 50 μg ml^−1^ RNase, and incubated for 2 h at 37 °C. A second extraction with chloroform:isoamilic alcohol (24:1) was performed, in which DNA was precipitated with 1/10 volume of sodium acetate and 2.5 volumes of ethanol, centrifuged, washed with ethanol 70 % (v/v) and resuspended in 50–100 μL of sterile distilled water.

#### 16S rDNA PCR amplification

PCR amplification of the complete 16S rRNA gene was carried out using the primers PA (5′-AGAGTTTGATCCTGGCTCAG-3′) and 907R (5′-CCGTCAATTCCTTTRAGTT 3′) (Muyzer et al. 1993; Massol-Deya et al. 1995). Reactions were performed in a final volume of 50 μL, containing 1X PCR buffer (Invitrogene), 200 μmol L^−1^ each of dATP, dCTP, dGTP and dTTP (Invitrogene), 2 mmol l^−1^ MgCl_2_, 5 μmol L^−1^ of each primer, 100 ng of genomic DNA and 1U of Taq DNA polymerase (Invitrogene). A Biometra thermocycler was used for amplification. The reaction consisted of an initial denaturation step at 94 °C for 5 min, followed by 35 cycles of 1 min at 94 °C, 1 min at 50 °C and 1 min at 72 °C and a final extension step of 5 min at 72 °C.

#### 16S rDNA sequencing and phylogenetic analysis

16S rDNA fragments amplified with primers PA and 907R from genomic DNA of isolates were purified with a JETquick spin column (Genomed, Germany) and sequenced with the same primers using a Beckman-Coulter automated DNA sequencer (model CEQ-2000) with dye terminators, following standard protocols and then identified using the BLAST tool of the National Center for Biotechnology Information (http://www.ncbi.nlm.nih.gov).

For DNA sequence analysis, alignments were made with ClustalX 2.1 and visually corrected with BioEdit Sequence Alignment Editor. The Neighbor Joining method was applied to estimate phylogenetic relationships among the isolates.

### Nitrogen fixation

In order to assess the nitrogen fixing capacity of the isolates, an assay based on the formation of a layer in semi-solid JNFb medium (without N) was carried out (Döbereiner et al. 1995). Bacteria were first grown in DYGS liquid media, then washed with saline solution (NaCl 0.85 %), and 20 µl of the culture were inoculated in triplicate in 10 mL vials containing 5 mL of semi-solid JNFb medium. The vials were stored at 30 °C for 7 days. After this period of time it was possible to observe a layer forming, characteristic of diazotrophic bacteria when in culture media. As a positive control, a wild bacteria was used (*Gluconacetobacter diazotrophicus* PAL 5, kindly supplied by Professor Fábio Lopes Olivares from the Universidade Federal Norte Fluminense).

### Assessment of antibiotic resistance by the colonies

Six antibiotics were used to test antibiotic resistance, at a concentration of 1000 µg/mL: chloramphenicol, ampicillin, metacicllin, erythromycin, canamycin and tetracycline. The colonies were inoculated into Nutrient Agar (NA) for 24 h at 28 °C. After growth, the colonies were transferred to a new agar plate with NA using a swab, and discs with 7 µl of each antibiotic were added. The plates were incubated at 28 °C for 24 h, after which the colonies were classified as either resistant or susceptible according to the halo formed around the antibiotic disc (Tawiah et al. 2012).

### Production of siderophores

The production of siderophores isolated from the bacterial colonies was assayed using the chrome azurol S (CAS) method as described by (Schwyn and Neilands 1987), with some modifications. The siderophores were produced in soy trypticase liquid media (STL) diluted (1:10) in 1 litre of deionised water. The CAS solution was prepared in a 100 mL volumetric flask with 6 mL of a 10 mM HDTMA solution in deionised water. Slowly stirring, 1.5 mL of an iron solution (1 Mm FeCl_3_·6H_2_O and 0.01 N HCl) and 7.5 mL of an aqueous solution of CAS (2 mM) were also added. Separately, 4.307 g of anhydrous piperazine was solubilized in approximately 20 mL of deionised water and 6.25 mL of concentrated HCl were added. The prepared buffer (pH 6.5) was transferred to the aforementioned volumetric flask and the volume was completed with 100 mL of deionised water. Bacteria were grown in a 50 mL Erlenmeyer flask containing 10 mL of a 1:10 STL diluted medium. The bacteria were then incubated at 28 °C for 24 h with a rotation of 160 rpm. 2 mL of the suspension were taken from the suspension aseptically and centrifuged at 12,000*g* for 10 min. 1 mL of CAS was then added to 1 mL of the resulting solution. The bacterial colonies that transformed the colour of the CAS from blue to yellow within 15 min were considered producers of siderophores (Louden et al. 2011). This assessment was carried out by using a qualitative method, therefore only the presence or absence of production was detected.

### Production of indole acetic acid (IAA)

The production of IAA was assessed using the colorimetric method described by (Gordon and Weber 1951), with a few modifications. Bacteria were grown in 10 mL of a nutritive broth at 28 °C for 24 h with constant agitation at 160 rpm before being transferred to 50 mL of the same medium to which tryptophan was added to make a final concentration of 2 and 200 µg/mL. At intervals of approximately 2 h (for a total period of 96 h), 3 mL (1 mL for the determination of the Optical Density (OD) and 2 mL for the determination of the production of IAA) of the broth were taken aseptically. In order to determine the production of IAA, samples were centrifuged for 10 min at 10,000×*g*. 1 ml of the supernatant of each sample was transferred to a test tube (10 mL) where 2 mL of Salkowski reagent (2 % FeCl_3_·6H_2_O and 37 % HCl_3_) were added. The test tubes were kept in the dark at 28 °C for 30 min, after which the presence of the hormone was established by the pink coloration and quantified through the spectrophotometer reading at 530 nm. Each strain was assessed in triplicate, with the negative control being constituted by the medium alone. The hormone’s production was predicted using a calibration curve obtained with 1, 2.5, 5, 10, 25, 50 and 100 μg mL^−1^ Acetic Indole Acid (Sigma^®^). Tryptophan was also tested at two doses, 2 (control) and 200 µg/mL, to evaluate the stimulation or inhibition of IAA production by isolates.

Pseudomonas fluorescens was included as a positive control PGPR strain known as producer of IAA (Oberhansli et al. 1991).

### Capacity for solubilizing distinct phosphate sources

The ability of bacterial isolates to solubilize various phosphate sources in solid NBRIP supplemented with Ca_3_(PO_4_)_2_ 5 gL^−1^ and phytic acid 1.6 gL^−1^ was evaluated. The plates containing the phosphate sources were inoculated with each bacterial isolate, by means of four equispaced bites on the surface of the medium, then further incubated at 28 °C for 15 days. The solubilizing capacity was evaluated based on the formation of halos around the colonies. The solubilization index (SI) was calculated using the ratio between the halo and colony diameters (Barraqueiro et al. 1976): IS = diameter halo/colony diameter.

### Antifungal effect

Interactions tests between the bacterial isolates and pathogenic fungi were carried out for six species of the latter: *Botrytis cinerea*, *Pestalotia* sp., *Alternaria* sp., *Phoma* sp., *Fusarium culmorum* and *Geotrichum candidum.*

Since bacteria and fungi have different growth rates, this assessment began by calculating the growth rates of the fungi in YEPGA medium (10 g of peptone, 10 g of yeast extract, 50 g of glucose and 15 g of agar), and based on this rate the bacterial incubation time was also determined. The YEPGA medium was chosen to allow the growth of both bacteria and fungi. Over four days the diameter of each fungus’ colony was measured (two perpendicular directions) (Broadbent et al. 1971). From the measurements, the expansion of the mycelium was calculated based on the linear equation diameter = (mycelium expansion x time) + y intercept, since fungi exhibit linear growth when on the surface of a petri dish with saturated nutritive conditions. After 2 days of fungal growth, the dishes received the inoculum of the bacteria, excepted for *B. cinerea* which demonstrated a slower growth rate; for this reason this bacteria were added after 7 days. Petri dishes were divided into 6 parts, with each of the fungi to be inoculated in the centre, and in each of the 6 parts a different strain of bacteria was added. A sector without any bacterial inoculation served as a negative control, and each bacteria was inoculated in triplicate. After the incubation period, the fungal growth was measured and photographed, then the resulting image was analysed using ImageJ, version 1.47^®^. This analysis allowed for determination of inhibition, by measuring the decrease in growth of each mycelium in the presence of bacteria in comparison with the negative control without bacteria.

### Statistical analysis

The experimental design was completely randomized with four repetitions. All analyses were validated by convenient residual analyses that did not show departure from the normal distribution according to the Kolmogorov–Smirnov test and showed homocedasticity among factors. The data were analyzed using ANOVA and means comparison were estimated values of Tukey´s test, when factors and interactions were significant. To compare the organic farming system against the conventional, we applied a *t* test for two independent samples and computed the 95 % confidence intervals for the corresponding mean difference. All statistical tests were performed using the statistical program IBM-SPSS V.22.

## Abbreviations

PGPR: plant growth-promoting rhizobacteria
N: nitrogen
P: phosphate
IAA: indole acetic acid
CFU: colony forming unit
O: organic
C: conventional
OD: optical density

## References

[CR1] Empresa brasileira de pesquisa agropecuária (EMBRAPA) (2005). Procedimento para coleta de amostra de solos. Ministério de agricultura pecuária e desenvolvimento. Agrobiologia 2005. Accessed from http://www.agencia.cnptia.embrapa.br/Repositorio/coleta_amostras_solo_000fhtbvqw702wyiv80v17a09ztd08zh.pdf. 15 Oct 2012

[CR2] Ahemad M, Khan MS (2010). Plant growth promoting activities of phosphate solubilizing *Enterobacter asburiae* as influenced by Fungicides. EurAsian J BioSci.

[CR3] Ahemad M, Khan MS (2011). Assessment of pesticide-tolerance and functional diversity of bacterial strains isolated from rhizospheres of different crops. Insight Microbiol.

[CR4] Ahemad M, Kibret M (2014). Mechanisms and applications of plant growth promoting rhizobacteria: current perspective. J King Saud Univ Sci.

[CR5] Ahemad F, Ahmad I, Khan MS (2008). Screening of free-living rhizospheric bacteria for their multiple plant growth promoting activities. Microbiol Res.

[CR6] Ahmed E, Holmström SJM (2014). Siderophores in environmental research: roles and applications. Microb Biotechnol.

[CR7] Amin SA, Hmelo LR, van Tol HM, Durham BP, Carlson LT, Heal KR, Morales RL, Berthiaume CT, Parker MS, Djunaedi B, Ingalls AE, Parsek MR, Moran MA, Armbrust EV (2015). Interaction and signalling between a cosmopolitan phytoplankton and associated bacteria. Nature.

[CR8] Araújo ASF, Leite LFC, Santos VB, Carneiro RFV (2009). Soil microbial activity in conventional and organic agricultural systems. Sustainability.

[CR9] Avis TJ, Gravel V, Antoun H, Tweddell RJ (2008). Multifaceted beneficial effects of rhizosphere microorganisms on plant health and productivity. Soil Biol Biochem.

[CR10] Azadeh BF, Sariah M, Wong MY (2010). Characterization of *Burkholderia cepacia* genomovar as a potential biocontrol agent of *Ganoderma boninense* in oil palm. Afr J Biotechnol.

[CR11] Barea JM, Pozo MJ, Azcón R, Azcón-Aguilar C (2005). Microbial co-operation in the rhizosphere. J Exp Bot.

[CR12] Barea AK, Pramanik K, Mandal B (2014). Response of biofertilizers and homo-brassinolide on growth, yield and oil content of sunflower (*Helianthus annuus* L. Afr J Agric Res.

[CR13] Barraqueiro FR, Baya AM, Cormenzana AR (1976). Establecimiento de índices para el estudio de la solubilizacion de fosfatos por bacterias del suelo. ARS Pharm.

[CR14] Bashan Y, de-Bashan LE, Prabhu SR, Hernandez JP (2014). Advances in plant growth-promoting bacterial inoculant technology: formulations and practical perspectives (1998–2013). Plant Soil.

[CR15] Beltrán-Esteve M, Reig-Martínez E (2014). Comparing conventional and organic citrus grower efficiency in Spain. Agric Syst.

[CR16] Bevivino A, Tabacchioni S, Chiarini L, Carusi MV, Del Gallo M, Visca P (1994). Phenotypic comparison between rhizosphere and clinical isolates of *Burkholderia cepacia*. Microbiology.

[CR17] Bevivino A, Sarrocco S, Dalmastri C, Tabacchioni S, Cantale C, Chiarini L (1998). Characterization of a free-living maize-rhizosphere population of *Burkholderia cepacia*: effect of seed treatment on disease suppression and growth promotion of maize. FEMS Microbiol Ecol.

[CR18] Bhardwaj D, Ansari MW, Sahoo RK, Tuteja N (2014). Biofertilizers function as key player in sustainable agriculture by improving soil fertility, plant tolerance and crop productivity. Microb Cell Fact.

[CR19] Bochner BR (2009). Global phenotypic characterization of bacteria. FEMS Microbiol Rev.

[CR20] Broadbent P, Baker KF, Waterworth Y (1971). Bacteria and actinomycetes antagonistic to fungal root pathogens in australian soils. Aust J Biol Sci.

[CR21] Buckling A, Wills MA, Colegrave N (2003). Adaptation limits diversification of experimental bacterial populations. Science.

[CR22] Caballero-Mellado J, Onofre-Lemus J, Estrada-de los Santos P, Martınez-Aguilar L (2007). The tomato rhizosphere, an environment rich in nitrogen-fixing *Burkholderia* species with capabilities of interest for agriculture and bioremediation. Appl Environ Microbiol.

[CR23] Chernin L, Ismailov Z, Haran S, Chet I (1995). Chitinolytic *Enterobacter agglomerans* antagonistic to fungal plant pathogens. Appl Environ Microbiol.

[CR24] Chiarini L, Bevivino A, Tabacchioni S, Dalmastri C (1998). Inoculation of *Burkholderia cepacia*, *Pseudomonas fluorescens* and *Enterobacter* sp. on *Sorghum bicolor:* root colonization and plant growth promotion of dual strain inocula. Soil Biol Biochem.

[CR25] Dai M, Hamel C, Bainard LD, Arnaud MS, Grant CA, Lupwayi NZ, Malhi SS, Lemke R (2014). Negative and positive contributions of arbuscular mycorrhizal fungal taxa to wheat production and nutrient uptake efficiency in organic and conventional systems in the Canadian prairie. Soil Biol Biochem.

[CR26] Davidson L (1988). Plant beneficial bacteria. Biotechnology.

[CR27] Dawwam GE, Elbeltagy A, Emara HM, Abbas IH, Hassan MM (2013). Beneficial effect of plant growth promoting bactéria isolated from the roots of potato plant. Ann Agric Sci.

[CR28] Di Cello F, Bevivino A, Chiarini L, Fani R, Paffetti D, Tabacchioni S, Dalmastri C (1997). Biodiversity of a *Burkholderia cepacia* population isolated from the maize rhizosphere at different plant growth stages. Appl Environ Microbiol.

[CR29] Döbereiner J, Baldani VLD, Baldani JI (1995). Como isolar e identificar bactérias diazotróficas de plantas não-leguminosas.

[CR30] Ferrara FIS, Oliveira ZM, Gonzales HHS, Floh EIS, Barbosa HR (2012). Endophytic and rhizospheric enterobacteria isolated from sugar cane have different potentials for producing plant growth-promoting substances. Plant Soil.

[CR31] Food and Agriculture Organization (FAO) (2012) FAO. FAOSTAT. http://faostat.fao.org/site/291/default.aspx. Accessed 30 June 2014

[CR32] Godfray HCJ, Beddington JR, Crute IR, Haddad L, Lawrence D, Muir JF, Pretty J, Robinson S, Thomas SM, Toulmin C (2010). Food security: the challenge of feeding 9 billion people. Science.

[CR33] Gordon SA, Weber RP (1951). Colorimetric estimation of indoleacetic acid. Plant Physiol.

[CR34] Grantina L, Kenigsvalde K, Eze D, Petrina Z, Skrabule I, Rostoks N, Nikolajeva V (2011). Impact of six-year-long organic cropping on soil microorganisms and crop disease suppressiveness. Žemdirb Agricult.

[CR35] Gupta M, Kiran S, Gulatic A, Singh B, Tewari R (2012). Isolation and identification of phosphate solubilizing bacteria able to enhance the growth and aloin-A biosynthesis of *Aloe barbadensis* Miller. Microbiol Res.

[CR36] Hayat R, Ali S, Amara U, Khalid Ahmed I (2010). Soil beneficial bacteria and their role in plant growth promotion: a review. Ann Microbiol.

[CR37] Huang GH, Tian HH, Liu HY, Fan XW, Liang Y, Li YZ (2013). Characterization of plant-growth-promoting effects and concurrent promotion of heavy metal accumulation in the tissues of the plants grown in the polluted soil by *Burkholderia* Strain LD-11. Int J Phytoremediation.

[CR38] Idriss EE, Makarewicz O, Farouk A, Rosner K, Greiner R, Bochow H, Richter T, Borriss R (2002). Extracellular phytase activity of *Bacillus amyloliquefaciens* FZB45 contributes to its plant-growth-promoting effect. Microbiology.

[CR39] Jackson LE, Burger M, Cavagnaro TR (2008). Roots, nitrogen transformations, and ecosystem services. Annu Rev Plant Biol.

[CR40] Jorquera MA, Hernández MT, Rengel Z, Marschner P, Mora ML (2008). Isolation of culturable phosphobacteria with both phytate-mineralization and phosphate-solubilization activity from the rhizosphere of plants grown in a volcanic soil. Biol Fertil Soils.

[CR41] Khan AA, Jilani G, Akhtar MS, Naqvi SMS, Rasheed M (2009). Phosphorus solubilizing bacteria: occurrence, mechanisms and their role in crop production. J Agric Biol Sci.

[CR42] Khan AL, Waqas M, Kang S-M, Al-Harrasi A, Hussain J, Al-Rawahi A, Al-Khiziri S, Ullah I, Ali L, Jung H-Y, Lee I-J (2014). Bacterial endophyte Sphingomonas sp. LK11 produces gibberellins and IAA and promotes tomato plant growth. J Microbiol.

[CR43] Laslo É, György É, Mara G, Tamás É, Ábrahám B, Lányi S (2012). Screening of plant growth promoting rhizobacteria as potential microbial inoculants. Crop Prot.

[CR44] Loaces I, Ferrando L, Scavino AF (2011). Dynamics, diversity and function of endophytic siderophore-producing bacteria in rice. Microb Ecol.

[CR45] Louden BC, Haarmann D, Lynne AM (2011). Use of blue agar CAS assay for siderophore detection. J Microbiol Biol Educ.

[CR46] Lugtenberg B, Kamilova F (2009). Plant-growth-promoting rhizobacteria. Annu Rev Microbiol.

[CR47] Madhaiyan M, Poonguzhali S, Hari K, Saravanan VS, Sa T (2006). Influence of pesticides on the growth rate and plant-growth promoting traits of *Gluconacetobacter diazotrophicus*. Pestic Biochem Physiol.

[CR48] Malboobi MA, Behbahani M, Madani H, Owlia P, Deljou A, Yakhchali B, Moradi M, Hassanabadi H (2009). Performance evaluation of potent phosphate solubilizing bacteria in potato rhizosphere. World J Microbiol Biotechnol.

[CR49] Marinari S, Lagomarsino A, Moscatelli MC, Di Tizio A, Campiglia E (2010). Soil carbon and nitrogen mineralization kinetics in organic and conventional three-year cropping systems. Soil Tillage Res.

[CR50] Marja R, Herzon I, Viik E, Elts J, Mänd M, Tscharntke T, Batáry P (2014). Environmentally friendly management as an intermediate strategy between organic and conventional agriculture to support biodiversity. Biol Conserv.

[CR51] Martínez-Aguilar L, Díaz R, Peña-Cabriales JJ, Estrada-de los Santos P, Dunn MF, Caballero-Mellado J (2008). Multichromosomal genome structure and confirmation of diazotrophy in novel plant-associated *Burkholderia* Species. Appl Environ Microbiol.

[CR52] Martins APL, Reissmann CB (2007). Material vegetal e as rotinas laboratoriais nos procedimentos químicoanalíticos. Scientia Agraria.

[CR53] Massol-Deya AA, Odelson DA, Hichey RP, Tiedje JM (1995) Bacterial community fingerprinting of amplified 16S and 16S–23S ribosomal DNA gene sequences and restriction endonuclease analysis (ARDRA). In: Akkermans ADL, van Elsas JD, de Bruijn FJ (eds) Molecular Microbial Ecology Manual. Kluwer Academic, Dordrecht, pp 3.3.2-1–3.3.2-8

[CR54] Mendonça V, Pedrosa C, Feldberg NP, Abreu NAA, Brito APF, Ramos JD (2006). Doses of nitrogen and simple superphosphate on papaya Formosa plant growth. Ciênc. Agrotec. Lavras.

[CR55] Moeskops B, Sukristiyonubowo Buchan D, Sleutel S, Herawaty L, Husen E, Saraswati R, Setyorini D, De Neve S (2010). Soil microbial communities and activities under intensive organic and conventional vegetable farming in West Java, Indonesia. Appl Soil Ecol.

[CR56] Muyzer G, de Waal EC, Uitterlinden AG (1993). Profiling of complex microbial populations by denaturing gradient gel electrophoresis analysis of polymerase chain reaction-amplified genes coding for 16S rRNA. Appl Environ Microbiol.

[CR57] Naher UA, Othman R, Panhwar QA (2013). Culturable total and beneficial microbial occurrences in long-term nutrient deficit wetland rice soil. Aust J Crop Sci.

[CR58] Nautiyal CS (1999). An efficient microbiological growth medium for screening phosphate solubilizing microorganisms. FEMS Microbiol Lett.

[CR59] Nery-Silva FA, Machado JC, Vilela de Resende ML, Lima LCO (2007). Inoculation methodology s of papaya fruits with fungi causing stem-end-rot. Ciênc. agrotec. Lavras.

[CR60] Oberhansli T, Defago G, Haas D (1991). Indole-3-acetic acid (IAA) synthesis in the biocontrol strain CHA0 of Pseudomonas fluorescens: role of tryptophan side chain oxidase. J Gen Microbiol.

[CR61] Patel KJ, Singh AK, Nareshkumar G, Archana G (2010). Organic-acid-producing, phytate-mineralizing rhizobacteria and their effect on growth of pigeon pea (*Cajanus cajan*). Appl Soil Ecol.

[CR62] Peeters C, Zlosnik JEA, Spilker T, Hird TJ, LiPuma JJ, Vandamme P (2013). *Burkholderia pseudomultivorans* sp. nov., a novel *Burkholderia cepacia* complex species from human respiratory samples and the rhizosphere. Syst Appl Microbiol.

[CR63] Peix A, Rivas R, Mateos PF, Martinez-Molina E, Rodriguez-Barrueco CE, Valazquez E (2003). *Pseudomonas rhizosphaerae* sp. nov., a novel species that actively solubilizes phosphate in vitro. Int J Syst Evol Microbiol.

[CR64] Pereg L, McMillan M (2014). Scoping the potential uses of beneficial microorganisms for increasing productivity in cotton cropping systems. Soil Biol Biochem.

[CR65] Perez PG, Ye J, Wang S, Wang XL, Huang DF (2014). Analysis of the occurrence and activity of diazotrophic communities in organic and conventional horticultural soils. Appl Soil Ecol.

[CR66] Pitcher DG, Saunders NA, Owen RJ (1989). Rapid extraction of bacterial genomic DNA with guanidium thiocyanate. Lett Appl Microbiol.

[CR67] Radíc T, Likar M, Hancevíc K, Bogdanovíc I, Paskovíc I (2014). Occurrence of root endophytic fungi in organic versus conventional vineyards on the Croatian coast. Agric Ecosyst Environ.

[CR68] Reed SC, Yang X, Thornton PE (2015). Incorporating phosphorus cycling into global modeling efforts: a worthwhile, tractable endeavor. New Phytol.

[CR69] Richardson AE, Barea JM, McNeill AM, Prigent-Combaret C (2009). Acquisition of phosphorus and nitrogen in the rhizosphere and plant growth promotion by microorganisms. Plant Soil.

[CR70] Rodríguez H, Fraga R (1999). Phosphate solubilizing bacteria and their role in plant growth promotion. Biotechnol Adv.

[CR71] Rodríguez H, Fraga R, Gonzalez T, Bashan Y (2006). Genetics of phosphate solubilization and its potential applications for improving plant growth-promoting bacteria. Plant Soil.

[CR72] Rogers A, McDonald K, Muehlbauer MF, Hoffman A, Koenig K, Newman L, Taghavi S, Lelie D (2011). Inoculation of hybrid poplar with the endophytic bacterium Enterobacter sp. 638 increases biomass but does not impact leaf level physiology. GCB Bioenergy.

[CR73] Santos-Villalobos S, Barrera-Galicia GC, Miranda-Salcedo MA, Peña- Cabriales JJ (2012). Burkholderia cepacia XXVI siderophore with biocontrol capacity against *Colletotrichum gloeosporioides*. World J Microbiol Biotechnol.

[CR74] Sarruge JR, Haag HP (1974). Análise química em plantas.

[CR75] Schwyn B, Neilands JB (1987). Universal chemical assay for the detection and determination of siderophores. Anal Biochem.

[CR76] Shannon D, Sen AM, Johnson DB (2002). A comparative study of the microbiology of soils managed under organic and conventional regimes. Soil Use Manag.

[CR77] Sharma SB, Sayyed RZ, Trivedi MH, Gobi TA (2013). Phosphate solubilizing microbes: sustainable approach for managing phosphorus deficiency in agricultural soils. Springer Plus.

[CR78] Singh RK, Malik N, Singh S (2013). Improved nutrient use efficiency increases plant growth of rice with the use of IAA-overproducing strains of endophytic *Burkholderia cepacia* strain RRE25. Microb Ecol.

[CR79] Singh P, Kumar V, Agrawal S (2014). Evaluation of phytase producing bacteria for their plant growth promoting activities. Int J Microbiol.

[CR80] Srinivas T, Sridevi M, Mallaiah KV (2008). Effect of pesticides on *Rhizobium* and nodulation of green gram *Vigna Radita* (L.) Wilczek. IUP J Life Sci.

[CR81] Suzuki MS, Zambolim L, Liberato JR (2007). Progress of fungal diseases and correlation with climatic variables in papaya. Summa Phytopathol.

[CR82] Tawiah AA, Gbedema SY, Adu F, Boamah VE, Annan K (2012). Antibiotic producing microorganisms from River Wiwi, Lake Bosomtwe and the Gulf of Guinea at Doakor Sea Beach, Ghana. BMC Microbiol.

[CR83] Trindade AV, Siqueira JO, Stürmer SL (2006). Arbuscular mycorrhizal fungi in papaya plantations of Espírito Santo and Bahia, Brazil. Braz J Microbiol.

[CR84] Trujillo I, Díaz A, Hernández A, Heydrich M (2007). Antagonismo de cepas de *Pseudomonas fluorescens* Y *Burkholderia cepacia* contra hongos fitopatógenos delarroz y el maíz. Rev. Protección Veg..

[CR85] Tsavkelova EA, Cherdyntseva TA, Netrusov AI (2005). Auxin production by bacteria associated with orchid roots. Microbiology.

[CR86] Unno Y, Okubo K, Wasaki J, Shinano T, Osaki M (2005). Plant growth promotion abilities and microscale bacterial dynamics in the rhizosphere of Lupin analysed by phytate utilization ability. Environ Microbiol.

[CR87] van Diepeningen AD, de Vos OJ, Korthals GW, van Bruggen AHC (2006). Effects of organic versus conventional management on chemical and biological parameters in agricultural soils. Appl Soil Ecol.

[CR88] Vassilev N, Vassileva M, Nikolaeva I (2006). Simultaneous P-solubilizing and biocontrol activity of microorganisms: potentials and future trends. Appl Microbiol Biotechnol.

[CR89] Zúñiga A, Poupin MJ, DonosoR Ledger T, Guiliani N, Gutiérrez RA, González B (2013). Quorum sensing and indole-3-acetic acid degradation play a role in colonization and plant growth promotion of arabidopsis thaliana by *Burkholderia phytofirmans* PsJN. Mol Plant Microbe Interact.

